# Prothrombotic state, endothelial injury, and echocardiographic changes in non-active sarcoidosis patients

**DOI:** 10.1038/s41598-022-25580-w

**Published:** 2022-12-09

**Authors:** Andzelika Siwiec-Kozlik, Piotr Kuszmiersz, Lukasz Kasper, Marzena Frolow, Pawel Kozlik-Siwiec, Teresa Iwaniec, Joanna Kosalka-Wegiel, Lech Zareba, Krzysztof Sladek, Jan G. Bazan, Stanislawa Bazan-Socha, Jerzy Dropinski

**Affiliations:** 1 Department of Rheumatology and Immunology, Jagiellonian University Medical College, Cracow, Poland; 2 Rheumatology and Immunology Clinical Department, University Hospital, Cracow, Poland; 3 Department of Internal Medicine, Jagiellonian University Medical College, Cracow, Poland; 4 Pulmonology and Allergology Clinical Department, University Hospital, Cracow, Poland; 5 Hematology Clinical Department, University Hospital, Cracow, Poland; 6 Department of Hematology, Jagiellonian University Medical College, Cracow, Poland; 7 Institute of Computer Science, College of Natural Sciences, University of Rzeszow, Rzeszow, Poland

**Keywords:** Respiratory tract diseases, Medical research, Coagulation system, Inflammation, Laboratory techniques and procedures, Echocardiography

## Abstract

Sarcoidosis is a multisystem inflammatory granulomatous disease of unknown cause that most commonly affects lungs and lymph nodes, with frequent yet asymptomatic cardiac involvement. The epidemiologically associated cardiovascular risk suggests an underlying prothrombotic state and endothelial dysfunction, currently understudied in the available literature. Therefore, we aimed to investigate prothrombotic plasma properties together with selected echocardiographic and laboratory biomarkers of cardiovascular injury in that disease. N = 53 patients with pulmonary sarcoidosis in clinical remission and N = 66 matched controls were assessed for inflammatory and endothelial injury biomarkers, plasma thrombin generation profile, and echocardiographic and lung function parameters. Sarcoidosis cases had impaired systolic and diastolic left ventricular function, higher concentrations of inflammatory markers, D-dimer and factor VIII activity compared to the controls. The coexistence of extrapulmonary disease was associated with elevated circulating vascular cell adhesion molecule 1, while cases with hypercalcemia had higher thrombomodulin concentration. Sarcoidosis was characterized by the unfavorably altered thrombin generation profile, reflected by the 16% higher endogenous thrombin potential (ETP), 24% increased peak thrombin concentration, and 12% shorter time to thrombin peak in comparison to the control group. ETP was higher in cases with proxies of pulmonary restriction, extrapulmonary–extracutaneous manifestation, and need for corticosteroids use. Despite the clinical remission, sarcoidosis is related to prothrombotic plasma properties and signs of endothelial injury, likely contributing to the higher risk of cardiovascular events. In addition, subclinical cardiac involvement may play an additional role, although further clinical and experimental studies are needed to verify these findings.

## Introduction

Sarcoidosis is a multisystem inflammatory disease characterized by the formation of microscopic, non-necrotizing structured masses named noncaseating granulomas. They are composed of macrophages, epithelioid cells, giant cells, and lymphocytes, predominantly CD4^+^ Th1 cells. The disorder most commonly affects the lungs and lymph nodes^1^. Albeit its etiology is unexplained despite decades of research efforts, studies have shown the role of genetic predisposition, environmental factors, and exposure to unknown infectious agents causing dysregulation of the immune response^2^.

Although sarcoidosis occurs most often in the nodal-pulmonary form, it can affect other organs, such as skin, eyes, heart, blood vessels, muscular, skeletal, and nervous systems, leading to their dysfunction. The extrapulmonary disease may be initially occult but finally, result in severe complications later in the disease course. In particular, cardiac sarcoidosis is diagnosed clinically in 2–5% of the patients, while autopsy reports indicate the frequency of cardiovascular involvement of about 20–30%; thus, the proper diagnosis is established postmortem^3^. Like the pulmonary disease, damage to the heart is caused by acute inflammation and/or tissue fibrosis^4^, potentially leading to progressive heart failure or death. A recent study indicates that the granulomas formation in the myocardium is related to the increased local expression of oncostatin M and the Reg3A and Reg3γ chemokines involved in the pathogenesis of chronic heart damage^5^. Since reversible inflammation results in irreversible tissue damage and remodeling, early diagnosis of cardiac involvement is essential for the patient outcome.

Venous thromboembolism (VTE), including deep vein thrombosis (DVT) and pulmonary embolism (PE), occurs at an incidence of about 1 per 1000 annually in the general adult population^6^. However, in sarcoidosis patients, the odds of VTE can be over three times higher (OR 3.35 [95% CI 3.25–3.46], after adjustment for age, sex and race)^7,8^. Furthermore, the involvement of the heart and blood vessels may increase thromboembolic events and sudden cardiac death in affected subjects^7,9^. It is estimated that cardiovascular complications account for 13–25% of fatal sarcoidosis cases in the United States. In Japan, where the cardiac form of the disease is more prevalent, this percentage is as high as 58%^3^.

The pathogenetic relationship between inflammation and coagulation is well established^10^. Inflammatory diseases are associated with an increased risk of thromboembolic complications^11^, as they contribute to Virchow’s triad: prothrombotic state, endothelial injury, and abnormalities of blood flow^12^. Inflammatory cytokines, such as tumor necrosis factor alpha (TNF-α) and interleukin (IL)-6, among others, may induce the expression of tissue factor (TF) on the monocytes^10^. Moreover, reduction of endothelial shear stress at inflammatory loci (as seen in decreased or turbulent blood flow) relates to endothelial proliferation, hypoxia, and release of P-selectin and von Willebrand factor (vWF) from the endothelium, promoting thrombus formation^13^. In turn, endothelial damage results in nitric oxide, thrombomodulin, and prostacyclin secretion, which to some extent may play a protective role on endothelium, in contrast to endothelin, constricting blood vessels^14^. Finally, research on sarcoidosis patients demonstrated reduced flow-mediated dilation of the brachial artery, associated with elevated circulating inflammatory biomarkers, such as TNF-α and intercellular adhesion molecule (ICAM)-1^15^. That observation points to endothelial damage, referring to systemic inflammation.

Consequently, acute inflammatory diseases (infections and rheumatologic disorders) are an established risk factor of VTE included in the widely-used Padua prediction score. Furthermore, two observational studies suggested sarcoidosis as an independent risk factor of PE, but did not differentiate between acute and chronic disease^9,16^. Several studies have previously analyzed the relationship between prothrombotic state, endothelial injury, and chronic inflammation in other lung diseases, including asthma^17^ and chronic obstructive pulmonary disease (COPD)^18^; however, no such data is available for sarcoidosis. Since blood coagulation is the common denominator of cardiovascular conditions and thromboembolism which can be modified using contemporary treatment options, its study in non-active sarcoidosis presents an essential scientific task with potential clinical applications.

Calibrated automated thrombin generation (CAT) assay is a well-established, universal, and reproducible tool to measure the entire kinetics of plasma thrombin formation, including its initiation, propagation, and termination^19^. In addition, Hemker et al. have shown that enhanced thrombin formation in that assay indicates a more extensive and faster activation of the blood coagulation cascade in vitro^20,21^. Moreover, further clinical studies have demonstrated that increased plasma thrombin generation is also an indicator of a higher risk of future VTE^22^, recurrent cardiovascular events^23^, or stroke in elderly patients^24^.

Here, we aimed to investigate the plasma thrombin generation profile in non-active sarcoidosis as a potential indicator of the prothrombotic state in these patients^25^. We have also analyzed its relationship with the inflammatory and endothelial injury biomarkers and lung function and echocardiographic parameters. According to our knowledge, such studies have not been performed to date.

## Methods

### Patients

We have conducted an observational, case–control comparative study of N = 53 white Caucasian adult patients with pulmonary sarcoidosis in clinical remission (n = 24, 43% women) who reported to our outpatient clinic in Cracow, Poland, from April 2019 to November 2019, and n = 66 matched controls.

The diagnosis of sarcoidosis and the extent of organ involvement was established by a physician according to the criteria of the American Thoracic Society/European Respiratory Society/World Association for Sarcoidosis and Other Granulomatous Disorders (ATS/ERS/WASOG)^26^. In each case, the diagnosis was confirmed by histological examination of hilar or mediastinal lymph node biopsy samples, demonstrating non-necrotizing granulomas in the absence of other etiologies (e.g., mycobacterial, parasitic or fungal infections, neoplasia, history of beryllium exposure). The samples were collected during endobronchial ultrasonography-guided transbronchial needle aspiration or mediastinoscopy. We have staged the disese by the traditional radiological criteria of intrathoracic changes^27^. Clinical remission was defined as stable lung function, chest radiograph findings, and lack of new respiratory or other disease-related symptoms over the past six months; maintenance therapy with low-dose glucocorticoid (7.5 mg per day or less of prednisone equivalent) or other immunosuppressants were permitted if the doses were not escalated over the past six months^28^.

Control subjects were enrolled from the hospital personnel and their acquaintances or relatives. They were frequency-matched with patients according to age, sex, body mass index (BMI), smoking status and comorbidities (i.e. arterial hypertension, hypercholesterolemia, and diabetes mellitus) to ensure no statistically significant differences between cases and controls were present.

The exclusion criteria for both the study and the control groups included: diagnosis of other autoimmune or pulmonary disease, any acute illness (including Löfgren's and Heerfordt's syndromes, trauma, infection), immobilization or surgical intervention within the past four weeks, pregnancy or breastfeeding, use of oral contraceptives or hormone replacement therapy, known thrombophilia, history of any thromboembolic event, such as myocardial infarction or stroke, coronary artery disease, congestive heart failure, left ventricular (LV) ejection fraction lower than 50%, atrial fibrillation, cancer, current anticoagulation treatment, chronic kidney disease (defined as estimated glomerular filtration rate [eGFR] < 60 ml/min/1.73 m^2^ using Modification of Diet in Renal Disease [MDRD] study formula) or liver injury (defined as liver enzymes elevated over twice the upper limit of normal range).

Subjects with arterial hypertension (defined as a history of blood pressure > 140/90 mmHg or treatment with antihypertensive medications), diabetes mellitus (defined as fasting serum glucose level > 7.0 mmol/l or therapy with anti-diabetic drugs), or hypercholesterolemia (defined as serum total cholesterol > 5.0 mmol/l or previously diagnosed and treated) were eligible.

The study design was approved by the Bioethics Committee of Jagiellonian University Medical College (protocol number 1072.6120.116.2019). The study was conducted according to the criteria set by the Declaration of Helsinki, and each subject signed informed consent before participating in the study.

### Lung function tests

Spirometry, whole-body plethysmography, and diffusing capacity for carbon monoxide were performed in sarcoidosis patients using a MasterLab “Jaeger” equipment following the ATS/ESR Statement^29^.

### Echocardiography

Transthoracic echocardiogram (TTE) was obtained from all patients and control individuals by standard protocols^30^. We used a GE Vivid 7 Dimension Ultrasound and a 4S 2–4 MHz probe with typical parasternal and apical views. Peak mitral inflow E and A velocity waves on pulsed-wave Doppler, E/A ratio, E-wave deceleration time, and isovolumic relaxation time were measured from the apical four-chamber view. In addition, the diastolic e’ velocity was obtained by tissue-Doppler imaging (TDI) at both the septal and lateral mitral origins on a four-chamber apical view. Furthermore, left ventricular (LV) filling indexes (E/e’ ratios) were calculated. LAVI (left atrial volume index) was calculated by dividing left atrial volume by body surface area.

### Laboratory investigations

Fasting blood samples were drawn between 7:00 and 11:00 a.m. using minimal stasis. Complete blood cell and platelet count, urea, creatinine, lipid profile, glucose, alanine aminotransferase, and fibrinogen were analyzed by routine laboratory techniques. The estimated glomerular filtration rate (eGFR) was calculated by the Chronic Kidney Disease Epidemiology Collaboration formula (CKD-EPI). C-reactive protein (CRP) was measured by the Cobas Integra System, while factor VIII activity (FVIII:C) by the one-stage clotting assay (Siemens, Marburg, Germany) and concentrations of D-dimer using the turbidimetric method (Innovance D-dimer, Siemens, Marburg, Germany).

Standardized high‐sensitivity immunoenzymatic assays (ELISAs) were used to measure serum levels of IL-6, VCAM-1, and soluble thrombomodulin (R&D Systems, Minneapolis, MN, USA). In plasma, thrombin-antithrombin complexes (TAT) were investigated by a commercially available immunoenzymatic test (Enzygnost TAT micro, Siemens, Marburg, Germany).

Moreover, blood samples were drawn into coagulation tubes filled with 0.109 mol/l (3.2%) buffered tri-sodium citrate solution (vol/vol 9:1), and centrifuged two times at 2500×*g* for 15 min, at room temperature, within 1 h of collection. The platelet-poor plasma was frozen in aliquots and stored at − 70 °C until the thrombin generation assay was analyzed.

Thrombin generation assay was applied using the calibrated automated thrombogram (CAT; Thrombinoscope BV, Maastricht, The Netherlands), according to the manufacturer’s instructions^19,21^. A reagent mixture of relipidated recombinant tissue factor (rTF) and phospholipids (20 μl, final concentration of 5 pmol/l and 4 mmol/l, respectively) was added to the platelet-poor plasma sample (80 μl), followed by the automatic addition of a fresh starting reagent containing calcium chloride (100 mmol/l) and a thrombin-specific fluorogenic substrate (Z-Gly-Gly-Arg-AMC) (2.5 mmol/l) in HEPES buffer. The reaction was completed in a microtiter well (Thermo Electron, Denmark). The fluorescence intensity was measured using the Fluoroscan Ascent® fluorometer (Thermo Fisher Scientific Oy, Vantaa, Finland) using the appropriate software (Thrombinoscope BV, version 3.0.0.29). All the experiments were run in duplicates.

**Figure 1:**
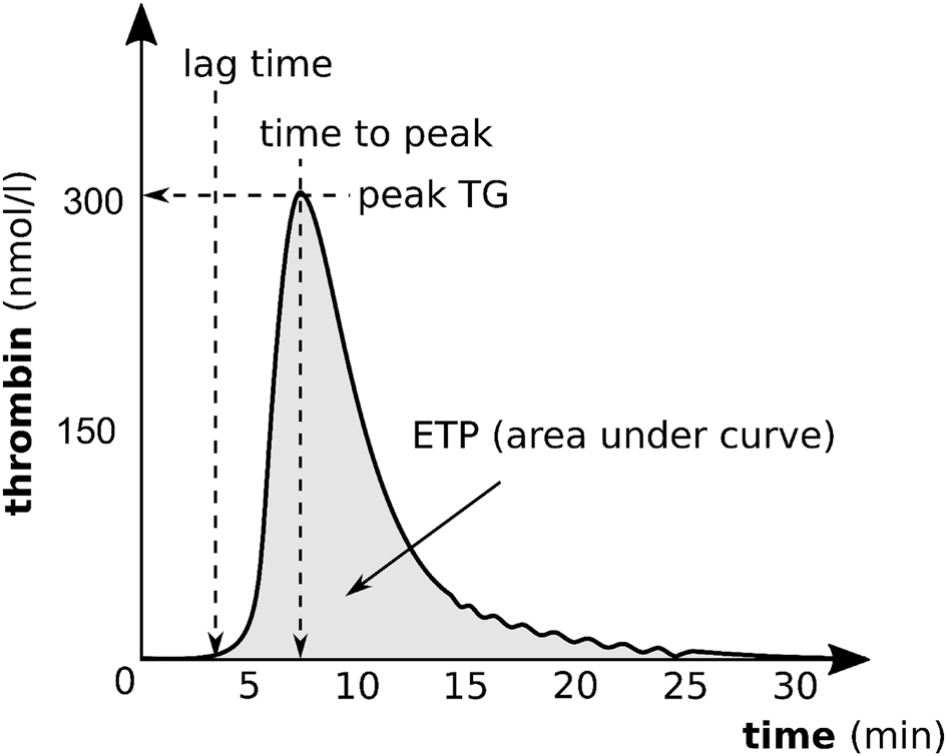
Thrombin generation curve (peak TG, peak thrombin generation; ETP, endogenous thrombin potential).

Four parameters of the thrombin generation curve were analyzed, as shown in Fig. 1.:

1. peak thrombin generation (peak TG, nmol/l)—the maximum concentration of thrombin formed during the time of registration,
2. endogenous thrombin potential (ETP, nmol/l × min)—the area under the curve showing thrombin formation,
3. lag time (min)—the time from the start of analysis until thrombin starts to generate,
4. time to thrombin peak (ttPeak, min)—the time from the start of thrombin generation until the maximum thrombin concentration is achieved.

### Statistical analysis

Statistical analysis was performed using the Statistica version 13.3 package for Microsoft Windows (StatSoft Inc., USA). Categorical variables were provided as percentages, and differences between study groups were analyzed using the χ2 test. The Shapiro–Wilk test assessed the normality of the continuous data distribution. As appropriate, they were presented as medians with a range between 25 and 75th percentiles or means with 95% confidence intervals (CIs) and compared using the Mann–Whitney U test, Kruskal–Wallis, or unpaired t-tests. A one-way analysis of covariance (ANCOVA) was applied to adjust for demographic confounders, i.e., sex, age, BMI, smoking habit, and comorbidities, i.e. arterial hypertension, diabetes mellitus, and hypercholesterolemia. A Spearman rank correlation test was performed to test the associations between continuous variables. Receiver Operating Characteristic (ROC) curves were analyzed to verify optimal cut-off values of ETP, peak TG, lag time, and D-dimer for calculation of odds ratios (ORs) with 95% CIs for differentiation between sarcoidosis and control cases in a multiple logistic regression model. Multiple linear regression models were utilized to indicate factors independently associated with ETP and peak TG. The results were considered statistically significant at a level of *p* < 0.05.

## Results

### Patients characteristics

Sarcoidosis and control individuals were well matched according to the demographic factors, including age, sex, BMI, smoking habit, and prevalence of comorbidities (Table 1). In Table 2 we have provided characteristics of sarcoidosis manifestation. As shown, the median duration of the disease was six (range 3–9) years, and the median age at diagnosis was 42 (range 35–50) years. In 11 (21%) patients, sarcoidosis was diagnosed incidentally in an asymptomatic stage due to abnormal chest X-rays obtained as a screening test for prophylactic purposes. The remaining subjects had constitutional signs, such as fatigue or weakness (n = 38, 71.7%), reduced exercise tolerance (n = 34; 64.2%), and pulmonary symptoms, including cough and dyspnea (n = 41, 77.4%). All patients had lung sarcoidosis, with radiological stage 2 in most cases (n = 42, 79.2%), i.e., hilar or mediastinal nodal enlargement and parenchymal changes. The most frequent extrapulmonary manifestations constitute skin changes (n = 12, 37.7%), extra-thoracic lymphadenopathy (n = 11, 20.8%), and splenomegaly (n = 7, 13.2%). The other rare manifestations included liver injury, nasal septum perforation, optic nerve involvement, and uveitis (Table 2). Interestingly, in 2 cases (3.8%), sarcoidosis occurred in the closest relatives in the past.

**Table 1:** A summary of demographic and laboratory characteristics of sarcoidosis patients and controls, the prevalence of internal medicine comorbidities.

	Patients n = 53	Controls n = 66	Difference *p*-value
Age, years	48 (40–58)	50.5 (44–58)	0.35
Male gender, n (%)	29 (55)	14 (21)	0.13
Body mass index, kg/m^2^	27.1 (24.7–30.4)	25.3 (24–27.9)	0.12
Hypertension, n (%)	24 (45)	22 (33)	0.38
Diabetes mellitus, n (%)	5 (9)	7 (11)	0.84
Hypercholesterolemia, n (%)	10 (19)	15 (23)	0.76
Smoking in the past, n (%)	27 (51)	22 (33)	0.2
Smoking in the past, packs/years	0 (0–8)	0 (0–1)	0.62
**Basic laboratory tests**			
White blood cells, 10^3^/μl	5.63 (4.82–7.26)	5.93 (4.82–6.75)	0.75
Neutrophiles, 10^3^/μl	3.32 (2.64–4.64)	3.35 (2.5–4.17)	0.5
Lymphocytes, 10^3^/μl	1.48 (1.05–1.88)	1.86 (1.58–2.26)	0.006*
Red blood cells, 10^6^/μl	4.91 (4.44–5.13)	4.41 (4.13–4.7)	0.001*
Hemoglobin, g/dl	14.4 (13.1–15.3)	13.1 (12.6–14)	0.01*
Blood platelets, 10^3^/μl	265 (220.5–303)	221.5 (198–277)	0.03*
Total cholesterol, mmol/l	5.1 (4.4–5.8)	5 (4.2–5.2)	0.4
High-density lipoprotein cholesterol, mmol/l	1.19 (1.02–1.48)	1.49 (1.34–1.82)	0.003*
Low-density lipoprotein cholesterol, mmol/l	3.39 (2.88–4)	3.21 (2.75–3.57)	0.3
Triglycerides, mmol/l	1.43 (1.16–2.23)	0.87 (0.7–1.45)	0.002*
Glucose, mmol/l	5 (4.7–5.6)	5.1 (4.3–6.1)	0.99
Creatinine, μmol/l	68 (62.1–85.3)	74 (68–87)	0.16
eGFR (CKD-EPI), ml/min/1.73m^2^	99.1 (90–106.9)	86.9 (74.1–97.5)	0.005*
C-reactive protein, mg/l	2.21 (1–5.3)	1.2 (1–2.6)	0.03*
Interleukin 6, pg/ml	2.14 (1.61–3.3)	1.79 (0.96–2.18)	0.03*
D-Dimer, ng/ml	384 (306–656)	232 (177–318)	< 0.001*
Factor VIII activity, %	151.4 (129.3–176.9)	116 (87.7–149.5)	< 0.001*
Fibrinogen, g/l	3.6 (3.1–4)	3.1 (2.4–3.6)	0.006*
**Endothelial injury biomarkers**			
Vascular cell adhesion molecule 1, ng/ml	897.8 (794.4–1025.8)	824.5 (671.2–956.6)	0.09
Thrombomodulin, ng/ml	4955 (4354–6034)	4636 (4020–5102)	0.09
**Thrombin generation profile in calibrated automated thrombogram**			
Endogenous thrombin potential, nmol/l × min	1767 (1508–1977)	1485 (1315–1667)	< 0.001*
Lag time, min	3 (2.67–3.33)	3 (2.49–3.39)	0.79
Peak thrombin generation, nmo/l	375.6 (336.5–417.3)	286.1 (234.6–323.6)	< 0.001*
Time to thrombin peak, min	5 (4.67–5.33)	5.67 (5.17–6.75)	< 0.001*
Thrombin-antithrombin complex, μg/l	3.77 (3.08–6.02)	3.62 (2.78–5.34)	0.21

Categorical variables are presented as numbers (percentages), continuous variables as means (with 95% confidence intervals) or as medians (and ranges between 25 and 75th percentiles), as appropriate. The results which are statistically significant are marked(*).

eGFR (CKD-EPI), estimated glomerular filtration (Chronic Kidney Disease Epidemiology Collaboration formula).

**Table 2:** Clinical characteristics of sarcoidosis patients (n = 53).

Clinical feature	
Age at diagnosis (years)	42 (35–50)
Duration of the disease (years)	6 (3–9)
Radiological stage of the disease, n (%)	
Stage 0	0
Stage 1	6 (11.3)
Stage 2	42 (79.2)
Stage 3	4 (7.5)
Stage 4	1 (1.9)
**Clinical manifestations (ever)**	
Fatigue/weakness, n (%)	38 (71.7)
Fever and low-grade fever, n (%)	21 (39.6)
Muscle weakness and reduced exercise tolerance, n (%)	34 (64.2)
Weight loss, n (%)	10 (18.9)
Arthralgia, n (%)	31 (58.5)
Respiratory symptoms (dyspnea, cough, chest pain), n (%)	41 (77.4)
Lymphadenopathy (except thorax), n (%)	11 (20.8)
Skin involvement, n (%)	12 (37.7)
Other organ involvement (except lungs and thorax), n (%)	6 (11.3)
Uveitis	2 (3.8)
Optic nerve	1 (1.9)
Nasal septum perforation	1 (1.9)
Liver injury	1 (1.9)
Splenomegaly, n (%)	7 (13.2)
Hypercalcemia/ hypercalciuria, n (%)	11 (20.8)
**Sarcoidosis therapy**	
Current oral glucocorticosteroids, n (%)	9 (17)
Systemic glucocorticosteroids therapy ever, n (%)	25 (47.2)
Systemic glucocorticosteroids therapy, years	3 (3–8)
Methotrexate treatment current or in the past, n (%)	2 (3.8)
**Spirometry**	
FEV_1_ before bronchodilator, L	3.27 (2.75–3.74)
FEV_1_ before bronchodilator, % of the predicted value	93.3 (82.5–106)
FEV_1_ after bronchodilator, L	3.18 (2.8–3.52)
FEV_1_ after bronchodilator, % of the predicted value	83.1 (67.5–102.2)
FVC before bronchodilator, L	4.14 (3.67–4.66)
FVC after bronchodilator, L	93.8 (83.2–100.4)
FEV_1_/VC (before bronchodilator)	88 (77.7–98)
FEV_1_/VC (after bronchodilator)	79 (74.8–88.6)
**Body plethysmography**	
Total lung capacity, L	5.99 (5.02–6.58)
Total lung capacity, % of the predicted value	91.9 (85.5–100.5)
Residual volume, L	1.61 (1.24–1.96)
Residual volume, % of the predicted value	78.5 (69–93.9)
**Gas exchange**	
Diffusing capacity or transfer factor of the lung for carbon monoxide (single breath), % of the predicted value	87.4 (79–93.8)

Categorical variables are presented as numbers (percentages), continuous variables as means (with 95% confidence intervals) or as medians (and ranges between 25 and 75th percentiles), as appropriate.

L, liters; FEV_1_, forced expiratory volume in 1 second; FVC, forced vital capacity; VC, vital capacity.

Almost half of the patients were treated with systemic corticosteroids with a median duration of 3 years (interquartile range 3–8 years), while only n = 9 (17%) received oral corticosteroids at the time of evaluation (Table 2). Two subjects (3.8%) were treated with methotrexate currently or in the past. Other immunosuppressants were not used in our patients.

Additionally, due to arterial hypertension, sarcoidosis patients were frequently treated with angiotensin-converting enzyme inhibitors or angiotensin receptor antagonists (n = 12; 37.7%), beta-blockers (n = 10; 18.9%), and diuretics (n = 7; 13.2%). Non-steroidal anti-inflammatory drugs or aspirin were applied by three (5.7%) of them.

Results of lung function tests in sarcoidosis patients are also provided in Table 2; most individuals had them within the normal range.

### Transthoracic echocardiographic parameters (Table 3)

**Table 3:** Baseline echocardiographic characteristics of sarcoidosis patients and controls.

	Patients n = 53	Controls n = 66	Difference *p*-value	Reference range
**LV basic parameters**				
LV ejection fraction, %	68 (65–68)	68 (68–70)	0.005*	52–72 (M), 54–74 (F)
LV end-diastolic dimension, mm	49 (46–52)	47 (44–50)	0.29	42–58 (M), 38–52 (F)
LV end-systolic dimension, mm	31 (29–34)	30 (29–31)	0.15	25–40 (M), 22–35 (F)
LV posterior wall thickness, cm	1 (0.8–1.1)	0.8 (0.8–1)	0.03*	0.6–1 (M), 0.6–0.9 (F)
Interventricular septum thickness, cm	1 (0.9–1.1)	0.9 (0.8–1)	0.04*	0.6–1 (M), 0.6–0.9 (F)
**LV diastolic function**				
MV E/A ratio, n	0.8 (0.7–1.3)	1.1 (0.8–1.3)	0.03*	0.8–2.0
MV E-wave deceleration time, ms	224 (190–248)	188 (178–198)	0.001*	140–240
Isovolumetric relaxation time, ms	110 (88–128)	83 (75–88)	< 0.001*	70–100
MV E/e’ ratio, n	7.8 (5.8–8.9)	6.6 (5.9–7.3)	0.03*	< 14
**RV basic parameters**				
RV mid diameter, mm	22 (21–23)	20 (19–23)	0.1	19–35
RV wall thickness, mm	4.6 (4–5)	4 (3.6–4.4)	0.002*	1–5
RV end-diastolic area, cm^2^/m^2^	10.4 (9.2–11.8)	8.9 (8–9.4)	0.006*	5–12.6 (M), 4.5–11.5 (F)
**RV diastolic function**				
TV E/A ratio, n	1 (0.8–1.2)	1.2 (1.1–1.3)	0.002*	0.8–1.2
TV E-wave deceleration time, ms	220 (190–246)	182 (180–210)	0.001*	120–230
Isovolumetric relaxation time, ms	68 (65–68)	64 (59–68)	0.001*	23–73
TV E/e’ ratio	6 (5–6.8)	5.6 (4.6–6.8)	0.79	2–6
**LA parameters**				
LA volume, ml	33 (32–37)	29 (27–31)	< 0.001*	18–58
LA area, cm^2^	21 (20–23)	18 (17–20)	< 0.001*	< 20
LAVI, ml/m^2^	18 (17–19)	16 (15–17)	< 0.001*	≤ 28
**RA parameters**				
RA volume, ml	30 (26–31)	28 (25–30)	0.38	18–58
RA area, cm^2^	18 (16–19)	17 (16–19)	0.51	≤ 18
**Assesment of pulmonary hypertension**				
Pulmonary artery systolic pressure, mmHg	34 (30–38)	26 (25–28)	< 0.001*	≤ 35
Tricuspid regurgitation velocity (TRV), m/s	2.8 (2.5–2.9)	2.3 (2.2–2.6)	< 0.001*	1.7–2.3
Pulmonary velocity acceleration time, ms	115 (100–129)	130 (124–144)	0.002*	> 130
Mean pulmonar arterial pressure, mmHg	34 (30–38)	26 (25–28)	< 0.001*	14–25

M, male; F, female; LV, left ventricle; RV, right ventricle; LA, left atrium; LAVI, left atrial volume index; RA, right atrium; MV, mitral valve; TV, tricuspid valve.

Transthoracic echocardiographic measurements revealed that sarcoidosis patients had larger dimensions of the right ventricle, thicker interventricular septum, and posterior LV walls (Table 3). They also had a slightly lower LV ejection fraction than controls. Interestingly, the two study groups also differed in the parameters of the diastolic cardiac function. For example, in sarcoidosis, we have documented prolonged isovolumic relaxation time in the left and the right ventricle, longer E-wave deceleration time (of mitral and tricuspid valve, respectively), and increased E/A and E/e’ ratio across the mitral valve. Additionally, we found several indices in the patient group suggesting a higher probability of pulmonary hypertension, such as increased pulmonary artery systolic and mean pressure, elevated tricuspid regurgitation velocity, and decreased pulmonary velocity acceleration time. Moreover, the left atrial volume index was greater in sarcoidosis than in controls (Table 3).

### Routine blood laboratory tests and endothelial injury biomarkers (Table 1)

Despite clinical remission, sarcoidosis was characterized by significantly higher concentrations of inflammatory markers, such as CRP, IL-6, and fibrinogen. These patients also had slightly elevated D-dimer, FVIII:C, red blood cell and platelet count, lower lymphocyte count, increased triglycerides, and high-density lipoprotein cholesterol levels (Table 1). Moreover, although the whole sarcoidosis group showed only a tendency towards higher concentrations of serum endothelial damage biomarkers, including thrombomodulin and VCAM-1 (*p* = 0.09, both), patients with the coexisting extrapulmonary manifestation, e.g., extra-thoracic lymphadenopathy, had elevated circulating VCAM-1 as compared to the remaining subjects (1295.7 [95% CI 954.9—1636.6] ng/ml, n = 11 vs. 882.1 [95% CI 835.3–928.8] ng/ml, n = 42; *p* < 0.001). Furthermore, those with documented prolonged hypercalcemia were characterized by increased thrombomodulin in peripheral blood (6305 [95% CI 5175–7436] ng/ml, n = 11, vs. 4997 [95% CI 4668–5327] ng/ml, n = 42; *p* = 0.002).

Concentrations of thrombomodulin and VCAM-1 correlated well with each other (r = 0.83, *p* = 0.003), and both of them were related to the plasma D-dimer (r = 0.85, *p* = 0.003 and r = 0.91, *p* < 0.001, respectively).

As expected, CRP was associated with IL-6 (r = 0.77, *p* < 0.001), which also remained in a positive relationship with plasma thrombomodulin (r = 0.78, *p* = 0.008). Furthermore, CRP and IL-6 were related to the D-dimer (r = 0.75, *p* = 0.012 and r = 0.69, *p* = 0.026, respectively).

### Thrombin generation assay

**Figure 2:**
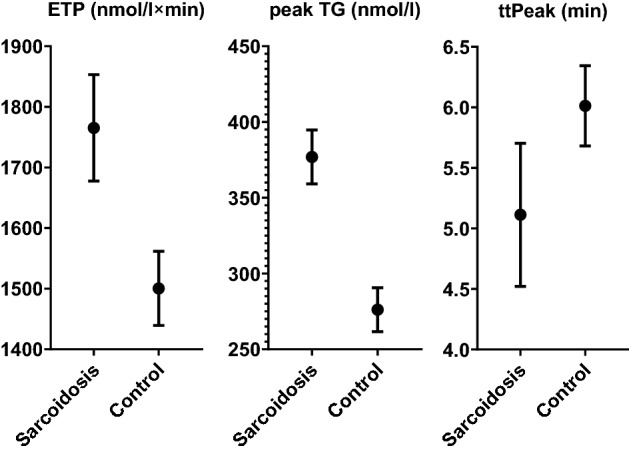
Differences in thrombin generation assay parameters between sarcoidosis patients and the control group. Mean value ± 95% confidence intervals are shown. *p*-value for all differences < 0.05.

In thrombin generation assay, sarcoidosis patients had an unfavorably altered thrombin generation profile, characterized by 16% higher ETP, 24% increased peak TG (*p* < 0.001 both, also after adjustment for potential confounders) and 12% faster ttPeak (*p* = 0.004 after adjustment for potential confounders) as compared to controls (Fig. 2.).

In sarcoidosis, we have documented higher odds of having elevated ETP and peak TG, given the following cut-off points: ETP elevation OR 2.92 [95% CI 1.9–4.51], for a cut-off of 1737 nmol/l × min, *p* < 0.001, peak TG elevation OR 5.73 [95% CI 3.2–10.25] for a cut-off of 352.7 nmol/l, *p* < 0.001.

Consequently, a predictive logistic regression model based on D-dimer, ETP, and ttPeak was able to discriminate between sarcoidosis patients and the control group with ROC AUC of 0.851 (AUC error of 0.0431) and cut-off points of 275 ng/ml, 1737 nmol/l × min, and 5.38 min, for D-dimer, ETP, and ttPeak, respectively (*p* < 0.0001 for that model).

Thrombin generation parameters were independent of demographic factors, including sex, age, and BMI in both studied groups. In turn, among basic laboratory measures, only triglycerides were linked with CAT assay variables, such as ETP and peak TG (r = 0.42, *p* = 0.03 and r = 0.52, *p* = 0.03, respectively).

Subgroup analysis revealed that neither radiological stage of the disease, symptoms at diagnosis, past inhaled corticosteroid, nor immunosuppressant use was related to the altered thrombin generation profile. In turn, ETP was higher in those with extrapulmonary–extracutaneous manifestation, including optic nerve, uvea, nasal septum and/or liver involvement (1986 [1887–2027] nmol/l × min, n = 6 vs. 1753 [1472–1921] nmol/l × min, n = 47; *p* = 0.02). The number of affected organs was not correlated with thrombin generation parameters.

Furthermore, patients on systemic corticosteroids, currently or in the past, were characterized by enhanced thrombin formation in CAT assay. The patients treated in the past had higher ETP as compared to those never treated (1872 [95% CI 1721–2022] nmol/l × min, n = 25 vs. 1670 [95% CI 1577–1764] nmol/l × min, n = 28; *p* = 0.02), while the patients using corticosteroids at assessment had elevated peak TG as compared to those currently untreated (420.7 [95% CI 354.9–486.5] nmol/l, n = 9 vs. 367.9 [95% CI 350.7–385.1] nmol/l, n = 44; *p* = 0.023).

Interestingly, in sarcoidosis, ETP was inversely correlated with total lung capacity (TLC, r = -0.46, *p* = 0.03) and vital capacity (VC) before bronchodilator (r = -0.32, *p* = 0.04). Consequently, ETP was significantly higher in the only four patients (7,5%) with TLC below the normal range, as compared to the remaining sarcoidosis individuals (2136 [95% CI 1283–2988] nmol/l × min vs. 1706 [95% CI 1557–1854] nmol/l × min, *p* = 0.04).

A multiple regression model (Table 4) showed that blood platelet count and serum IL-6 were independently and positively associated with ETP. Likewise, platelet count and FVIII:C remained in a positive relationship with peak TG. Furthermore, both analyzed thrombin generation variables were independently related to thrombin-antithrombin complexes concentration (positively) and eGFR (negatively). However, those variables explained only 32% and 43% of the ETP and peak TG variability.

**Table 4:** Multiple linear regression models for a relative increase of endogenous thrombin potential and peak thrombin generation in sarcoidosis patients.

	β (95%CI)	R^2^	F	*p*
**Endogenous thrombin potential (ETP; nmol/l × min)**				
Thrombin–antithrombin complex (µg/l)	0.32 (0.18–0.45)	0.32	4.28	0.006
Interleukin 6 (pg/ml)	0.32 (0.19–0.46)			
Blood platelet count (× 10^3^/µL)	0.29 (0.15–0.43)			
Estimated glomerular filtration rate (ml/min/1.73m^2^)	− 0.16 (− 0.30 to − 0.03)			
**Peak thrombin generation (peak TG; nmol)**				
Factor VIII activity (%)	0.33 (0.19–0.46)	0.43	6.72	< 0.001
Thrombin–antithrombin complex (µg/l)	0.37 (0.24–0.50)			
Blood platelet count (× 10^3^/µL)	0.18 (0.05–0.31)			
Estimated glomerular filtration rate (ml/min/1.73 m^2^)	− 0.16 (− 0.29 to − 0.03)			

The resulting standardized regression coefficient (β) with 95% confidence interval (95%CI) for a factor (independent variable) indicates the increase/decrease in standard deviations (SDs) of a dependent variable (ETP or peak TG), when that particular factor increases with 1 SD and all other variables in the model remain unchanged.

Regarding echocardiographic measures, we found a positive association between ETP and systolic pulmonary artery pressure (β = 0.26 [95% CI 0.12–0.4], *p* = 0.04, after adjustment for potential confounders).

## Discussion

In the present study, we have shown for the first time that sarcoidosis is characterized by an unfavorably altered thrombin generation profile, reflected by the higher ETP and peak TG and extended ttPeak as compared to well-matched controls. Thus, our results provide evidence for a prothrombotic state in clinically stable, non-active pulmonary sarcoidosis, which is consistent with previous epidemiological cohort studies^7,9,16^ and systematic reviews^8,31^ demonstrating an increased risk of cardiovascular events in this disorder.

Interestingly, higher serum IL-6, blood platelet count, and FVIII activity were independently associated with increased thrombin formation in sarcoidosis. At the same time, demographic factors, such as age, gender, and BMI, had no impact. Indeed, IL-6 is a robust prothrombotic agent, increasing TF expression on monocytes and endothelial cells and promoting their activation. Furthermore, IL-6 may enhance the production of fibrinogen, FVIII, and platelets^32^, which secrete proinflammatory and procoagulant mediators, such as P-selectin, platelet-activating factor, and thromboxane after activation^33^. Likewise, elevated FVIII may promote thrombin formation in response to inflammation or endothelial injury, further enhancing the prothrombotic state^34^.

Our results mirror previously published data on increased thrombin generation in other chronic inflammatory diseases, such as asthma^35^, rheumatoid arthritis^36^, systemic sclerosis^37^, systemic lupus erythematosus^33^ and autoimmune inflammatory myopathies^38^. They all point to the complex nature of the coagulation pathway control; however, inflammation and endothelial damage were essential factors associated with the prothrombotic state in most of them. On the contrary, discretion concerns demographic factors, such as age and BMI, which were predictors of CAT assay parameters, e.g., in asthma^35^. This discrepancy suggests that in sarcoidosis, the role of inflammatory response is much more significant^39,40^ and outweighs other less critical variables. Furthermore, our results indicate that even in a clinically non-active disease, the local or low-grade systemic inflammation may contribute to the prothrombotic plasma properties with potential clinical outcomes. Moreover, the patients with a more severe form of the disease, e.g., with lung function changes or requiring systemic corticosteroids, might be at a higher risk of thromboembolic complications. However, chronic exposure to corticosteroids poses increased cardiovascular risk and could be an independent variable contributing to the observed cardiovascular changes^41^.

Our results do not explain how lung inflammatory granulomas drive the prothrombotic state. One possible explanation might be related to the activity of Rac-1, a small G-coupled protein, a molecular switcher regulating cell adhesion, proliferation, migration, and chemotaxis. It has been demonstrated that Rac-1 implies T-cell and macrophage to sarcoid granulomas formation^42^. Furthermore, a murine sepsis model provides evidence that it also activates a coagulation pathway^43^. In turn, generated active coagulation factors, such as thrombin, may aggravate local or systemic inflammation by protease-activated receptors (PARs) on inflammatory, epithelial, and endothelial cells in the lungs. PARs are triggered by several proteases, including neutrophil elastase, granzymes of cytotoxic lymphocytes, and thrombin, enhancing local inflammatory response^44^. Moreover, it has been documented that activating PAR-1 on monocytes and macrophages upregulates the production of oncostatin M, a pleiotropic cytokine implicated in the pathology of the heart and vascular damage^45^.

As expected, increased ETP was related to the higher serum triglycerides. However, in our study, that association was more potent than previously reported in the general population^46^. Therefore, it may underline the importance of strict dyslipidemia management in sarcoidosis patients. In turn, the inverse relationship between thrombin generation and kidney function is well-known and mirrors other reports^47,48^.

Another important issue that merits comment is a higher circulating VCAM-1 in sarcoidosis patients with extrapulmonary lymphadenopathy. VCAM-1 is an endothelial cell biomarker, which appears on these cells after activation. Therefore, its higher blood concentration might suggest endothelial injury. However, it has been previously shown that increased VCAM-1 on endothelial cells is the first step of signaling mechanisms in lymph nodes, leading to lymphocyte accumulation and lymph nodes enlargement^49^. Therefore, one may speculate that increased VCAM-1 in our study does not necessarily indicate vascular damage. Instead, its higher level, interestingly related to the lower lymphocyte count in peripheral blood (data not shown), may reflect vascular susceptibility to the lymphocyte homing into the sarcoid lymph nodes.

In turn, the second analyzed endothelial damage biomarker, thrombomodulin, was higher in patients with hypercalcemia. Hypercalcemia affects about 20% of sarcoidosis individuals. The majority of those cases can be explained by the overproduction of 1,25(OH)_2_D_3_ by activated macrophages^50^. Of note, the active form of vitamin D3 stimulates osteoblasts to synthesize thrombomodulin^51^. Thus, higher thrombomodulin in hypercalcemia might also be related to the macrophage stimulation in sarcoid granulomas.

The last issue for discussion in our data is echocardiographic findings. In comparison to the controls, the patient group had enlarged dimensions of the right ventricle, left atrium, and thicker LV walls. Furthermore, sarcoidosis was related to diastolic cardiac dysfunction and the increased probability of pulmonary hypertension. Apart from cardiological cases, diastolic LV impairment is often demonstrated in pulmonary patients, particularly those with COPD or sleep apnea syndrome^52,53^. It is related to heart wall relaxation disorders, LV mass hypertrophy, and myocardial fibrosis, leading to increased LV filling pressure, left atrium pressure, and, consequently, pulmonary hypertension, which ultimately causes clinical symptoms^54^. Additionally, a postulated mechanism underlying cardiac diastolic failure is endothelial dysfunction of the coronary microcirculation due to increased circulating inflammatory cytokines (e.g., IL-6, TNF-α, pentraxin-3)^55,56^. Furthermore, coronary circulation's endothelial damage likely induces cardiomyocyte hypertrophy and increased collagen production in interstitial tissue^57^.

Documented echocardiographic features seem clinically insignificant; however, they might increase the risk of cardiovascular events in sarcoidosis. Moreover, the elevated left atrial volume index has been shown as an independent predictor of adverse cardiovascular outcomes^58^. While cardiac sarcoidosis is asymptomatic in 95% of the affected patients, untreated inflammation and progressive myocardial fibrosis can lead to heart chamber abnormalities and dysfunction. It is possible that the described diastolic dysfunction may be a consequence of undetected cardiac sarcoidosis. Of note, the diagnosis of this abnormality is difficult. Identified electrocardiographic or echocardiographic findings can often be explained by comorbidities such as hypertension or coronary heart disease^3^. At the same time, the sensitivity and specificity of the standard two-dimensional (2D) echocardiography are limited. However, new techniques, such as speckle tracking echocardiography, cardiac magnetic resonance, and positron emission tomography, can be beneficial in the early detection of inflammatory and fibrotic changes of the heart and thus need to be recommended in patients with suspected cardiac sarcoidosis^59–61^.

### Study limitations

Our study has several limitations. First, the study sample was relatively small; in particular, subgroup analysis should be interpreted with caution. Second, laboratory measures were analyzed once, and we cannot exclude their changes over time. Moreover, our observational study has no follow-up; thus, we cannot verify if enhanced plasma thrombin generation refers to the increased risk of VTE episodes. Third, sarcoidosis patients had internal comorbidities that may affect thrombin generation assays. However, we have matched controls for them and adjusted statistics in the ANCOVA analysis. Thus, we believe that the primary outcomes presented here are reliable. Also, the effect of the medication used was beyond the scope of our study. However, higher peak TG in patients currently treated with systemic corticosteroids is an interesting finding, possibly related to those medications' unfavorable side effects, as previously shown in healthy individuals^62^. Therefore, sarcoidosis patients on systemic corticosteroids might be at increased risk of VTE^63^. Finally, we did not determine other potential modulators of coagulation, e.g., genetic variants. Given prothrombotic abnormalities in circulating blood of first-degree relatives of patients with venous thrombosis^64^, we cannot exclude that a documented prothrombotic state is genetically determined, at least to some extent.

## Conclusions

The current study is the first to show increased in vitro plasma thrombin generation in patients with sarcoidosis, which might indicate the prothrombotic state in that disease. However, further extensive observational and prospective studies are needed to verify and explore that topic.

## Data Availability

The datasets used and analysed during the current study are available from the corresponding author on reasonable request.
